# Arsenic-Induced Injury of Mouse Hepatocytes through Lysosome and Mitochondria: An In Vitro Study

**DOI:** 10.1155/2022/1546297

**Published:** 2022-09-08

**Authors:** Amal Santra, Debasree Bishnu, Suman Santra, Subhadip Ghatak, Partha Sarathi Mukherjee, Gopal Krishna Dhali, Abhijit Chowdhury

**Affiliations:** ^1^JCM Centre for Liver Research and Innovations, Kolkata, India; ^2^Liver Foundation, Kolkata, West Bengal, India; ^3^Centre for Liver Research, School of Digestive & Liver Diseases, Institute of Post Graduate Medical Education & Research, Kolkata, India

## Abstract

**Background and Aims:**

The cellular mechanism of liver injury related to arsenic toxicity is ill defined. It is thought that oxidative stress and mitochondrial dysfunction may play some role in arsenic-induced liver damage. In this study, we evaluated subcellular events within the primary cultured mouse hepatocytes when exposed to inorganic arsenic.

**Methods:**

Primary cultured mouse hepatocytes were treated with 10 *μ*M arsenic for different time periods. Reactive oxygen species (ROS) formation, functional changes of the lysosome and mitochondria, and mode of hepatocytes death were studied by laser confocal microscopy, fluorescence spectroscopy, and flow cytometry. Expression of proapoptotic member of the BCL-2 family of genes BAX and antiapoptotic BCL-2 mRNA expression were studied by real-time PCR. Cytochrome c expression was studied by Western blotting.

**Results:**

Fluorescence spectroscopy as well as flow cytometric analysis revealed that arsenic-induced formation of ROS was time dependent. Confocal microscopy showed initiation of ROS formation from periphery of the hepatocytes at 30 min of arsenic exposure that progressed to central part of the hepatocytes at 3 h of arsenic exposure. The ROS formation was found to be NADPH oxidase (NOX) dependent. This low level of intracellular ROS induced lysosomal membrane permeabilization (LMP) and subsequently released cathepsin B to the cytosol. The LMP further increased intracellular ROS which in turn triggered induction of mitochondrial permeability transition (MPT). Pretreatment of hepatocytes with LMP inhibitor bafilomycin A (BafA) significantly decreased, and LMP inducer chloroquine (ChQ) significantly increased the production of ROS suggesting that LMP preceded enhanced ROS generation in response to arsenic. MPT was accompanied with increase in BAX : BCL2 mRNA ratio resulting in upregulation of caspase 3 and increased hepatocyte apoptosis.

**Conclusion:**

Although arsenic-related oxidative liver injury is well established, neither the site of origin of ROS nor the early sequence of events in arsenic toxicity due to ROS is known. We believe that our study provides evidences elucidating the early sequence of events that culminates in the death of the mouse hepatocytes during arsenic exposure.

## 1. Introduction

Arsenic is a natural component of the earth's crust that poses a serious environmental concern worldwide. Primarily due to geological changes, groundwater arsenic contamination places millions of people at risk of significant morbidity and mortality related to long-term exposure, mainly in drinking water [1]. Arsenic is long recognized to be a cellular toxin. The manifestations of chronic exposure to arsenic are protean, involving nearly every organ system of the body, in neoplastic as well as nonneoplastic vascular and fibrotic disorders [2, 3]. Chronic liver disease, represented histologically and clinically as portal fibrosis, structural disarray, and portal hypertension, has been recognized to be an important feature of chronic arsenicosis [4–7].

It is now well recognized that generation of reactive oxygen species (ROS) contributes to the initiation and switching of progression in chronic liver diseases of different etiology [8]. This has been very well described in nonalcoholic fatty liver disease (NAFLD) and chronic hepatitis C virus infection (HCV) [9, 10]. Enhanced ROS formation in hepatocytes causes perturbation in the structural and functional stability in the hepatic microenvironment thereby causing hepatocyte apoptosis and hepatic stellate cell (HSC) activation that are of relevance in the pathogenesis of fibrotic liver injury [11, 12].

Liver is the primary site of biotransformation and metabolism of inorganic arsenic and generates ROS during the process of its metabolism [13]. There are considerable evidences that ROS are involved in the cytotoxicity of arsenic in different tissues. Integrity of mitochondria is important in cell survival [14]. Mitochondrial permeability transition (MPT) is an important event that leads to alterations of mitochondrial energy metabolism, leading to ROS generation and oxidative stress, which spread the fire to other subcellular organelles, including the nucleus, resulting in cell injury and death [15]. It has also been suggested that lysosomes and the lysosomal proteases such as cathepsin B and D also act as proapoptotic mediators of apoptosis, in addition to mitochondrial release of cytochrome c and the activation of the caspase family of proteases [16]. While liver cell injury in arsenicosis is fairly well studied clinically, the temporal and spatial sequence of subcellular changes and the contribution of ROS in the process have not been studied in details.

In the present study, we have tried to elucidate sequences of events during arsenic exposure within the primary cultured mouse hepatocytes to delineate the crosstalk between lysosome and mitochondria in the execution of liver cell injury in arsenic toxicity.

## 2. Methods and Materials

### 2.1. Mouse Strain

Male BALB/c mice procured from the National Center for Laboratory Animal Sciences (NCLAS), Hyderabad, India, were used for primary hepatocyte isolation. The study was approved by the institutional animal ethical committee (IAEC/AS-2/2006/UCM-51), and all experiments were conducted in compliance with the said committee.

### 2.2. Isolation of Mouse Hepatocytes and Treatment with Arsenic

Hepatocytes were isolated from 25 to 30 g overnight-fasted male BALB/c mice by collagenase perfusion as described previously [17]. Hepatocytes were resuspended in William's E medium containing 2 mM L-glutamine, 10% fetal bovine serum (FBS), 100 nM insulin, 100 nM dexamethasone, 100 U/mL penicillin, and 100 *μ*g/mL streptomycin. Cell viability was greater than 97%, as determined by trypan blue exclusion test. After primary culture, the viable cells were seeded at a density of 1 × 10^6^ cells in William's E medium without FBS. Hepatocytes were treated with arsenic in FBS free media at a final concentration of 10 *μ*M for different time periods to study intracellular ROS, lysosomal integrity study, MPT, and cell death. All cell culture reagents and drugs were procured from Sigma Aldrich, St. Louis, MO, USA.

### 2.3. Treatment Schedule of Different Inducers and Inhibitors

In an experiment involving ROS inhibitor, mouse primary cultured hepatocytes were treated with 10 *μ*M NADPH oxidase inhibitor, diphenyliodonium (DPI) (Sigma Aldrich, St. Louis, MO, U.S.A) 30 min before arsenic treatment was initiated. For induction and inhibition of lysosomal membrane permeabilization (LMP), in other set of experiments, cells were treated either with 20 *μ*M of chloroquine (ChQ) (Sigma Aldrich, St. Louis, MO, U.S.A) or with 10 *μ*M of bafilomycin A (BafA) (Sigma Aldrich, St. Louis, MO, U.S.A) in serum-free medium 30 min prior to arsenic treatment. For inhibition of MPT, in another set of experiments, cells were treated with 10 *μ*M cyclosporin A (CsA; Sigma Aldrich, St. Louis, MO, U.S.A) in serum-free William's E medium for 30 min, followed by appropriate treatment with arsenic.

### 2.4. Detection of Intracellular ROS Formation

Intracellular ROS were assessed using 5 *μ*M fluorescent probe 2′, 7′-dichlorofluorescin diacetate (DCF-DA) [18] of the primary cultured mouse hepatocytes with or without arsenic treatment at different time points using multimode plate reader (Tecan Genios; U.S.A) and flow cytometer (BD FACS Calibur, BD Bioscience, Pharmingen, U.S.A.) using FL1 channel to detect DCF fluorescence of gated viable cells and confocal microscope (Leica; TCS SPE). At the end of the indicated time periods, cells were washed in phosphate-buffered saline (PBS), trypsinized, and resuspended in PBS. For quantification of intracellular ROS, cells were lysed using 0.1% Triton X-100, and the fluorescence intensity of DCF in the cell lysate was determined with multimode plate reader at excitation and emission wavelengths of 488 nm and 535 nm, respectively. For flow cytometric analysis, cells were washed once with PBS and then placed in tubes containing PBS containing 0.1% BSA and 0.01% azide from 10,000 cells in flow cytometer using Cell Quest software using FL1 channel. For visualization of intracellular ROS within the hepatocytes, cells were placed on microscopic slides, and green fluorescence of DCF was observed by laser confocal microscope with an excitation wavelength of 488 nm and emission wavelength of 535 nm.

### 2.5. Lysosomal Integrity Assay by Acridine Orange (AO) Relocation Method

In order to perform lysosomal integrity assay, primary cultured mouse hepatocytes were first preincubated with 5 *μ*g/mL acridine orange (AO) solution (Sigma Aldrich, St. Louis, MO, U.S.A) in William's E medium for 20 min in dark at 37°C [19]. Following staining, the cells were rinsed twice with PBS to remove the excess stain and treated with or without 10 *μ*M arsenic for different time points. At the end of treatment, the cells were mounted on a glass slide with a cover slip using antifade mounting medium (Sigma Aldrich, St. Louis, MO, U.S.A) and observed under the confocal microscope using excitation wavelength of 488 nm and emission wavelength of 535 nm.

### 2.6. Cathepsin B Activity Assay

Whole cell lysate was prepared using lysis buffer provided in assay kit (K140-100; Biovision). Cathepsin B activity assay was determined fluorometrically according to manufacturer's instructions in cell lysate using the cathepsin B activity assay kit (K140-100; Biovision). Fluorescence was measured in fluorescence spectrophotometer with an excitation wavelength of 400 nm and an emission wavelength of 505 nm.

### 2.7. Detection of Mitochondrial Permeability Transition

To monitor the onset of mitochondrial permeability transition (MPT), primary cultured mouse hepatocytes were seeded in culture medium at 37°C with 0.5 *μ*mol/L tetramethylrhodamine methyl ester (TMRM; Sigma Aldrich, St. Louis, MO, U.S.A) for 15 min followed by 1 *μ*mol/L calcein (Sigma Aldrich, St. Louis, MO, U.S.A) for another 15 min [20]. TMRM is a membrane permeable cationic fluorophore that accumulates electrophoretically into mitochondria in response to their negative potential; however, calcein (623 d) is unable to enter mitochondria because of its impermeability of the mitochondrial inner membrane and is distributed exclusively in the cytosolic space. Redistribution of the green fluorescence of calcein from cytosol into mitochondria signifies the onset of MPT, and the decrease of red fluorescence of TMRM in mitochondria indicates leakage of mitochondrial membrane. After seeding, the cells were washed twice with Krebs-Ringer-Hansleit (KRH) buffer and incubated at 37°C in William's E medium with arsenic before mounting on the microscope stage. Monitoring the fluorescences of TMRM and calcein were carried out in time sequences.

### 2.8. Annexin V and Propidium Iodide Staining and Flow Cytometry for Detection of Apoptosis

After specified time point of arsenic treatment, 1 × 10^5^ cells/mL were fixed with cold methanol-acetic acid and stained with propidium iodide (PI) and Annexin V using Annexin-V-Fluos staining kit (Roche, Germany) for 15 min as per protocol of the manufacturer. The fluorescence of Annexin V-positive cells and PI-positive cells was detected from 10,000 cells by flow cytometry (BD FACS Calibur, BD Bioscience, Pharmingen, U.S.A.) using FL1 channel (for fluorescein-labeled Annexin V) and FL2 channel (for PI), respectively. Control experiments without arsenic treatment were carried out concurrently.

### 2.9. Caspase 3 Activity Assay

Caspase-3 activity was carried out in cell lysates using a specific fluorometric assay kit (Catalog # K105-100; BioVision, U.S.A) as per the manufacturer's instructions. Briefly, at the end of arsenic treatment schedules, hepatocytes were washed twice with cold PBS, and 2 × 10^6^ hepatocytes were resuspended in 200 *μ*L chilled lysis buffer provided in the kit and kept in ice for 10 min. Cell lysate was prepared by centrifugation at 14,000 rpm for 20 min. For caspase 3 activity assay, in flat bottom black 96-well plate, 50 *μ*L cell lysate and 50 *μ*L reaction buffer containing 10 mM DTT were added in each well. Finally, 5 *μ*L of 1 mM DEVD-AFC was added to each well and incubated the plate at 37°C for 2 h. At the end of incubation period, fluorescence reading of the plate was measured using a multimode plate reader (Spectra Max i3X; Molecular Devices; San Jose, CA) equipped with 400 nm excitation filter and 505 nm emission filter. Results were expressed as folds of increase of the control experiments.

### 2.10. Preparation of Cell Lysate

Whole cell lysate was prepared using RIPA buffer (Cell Signaling Technology, U.S.A.) with protease inhibitor cocktail (Roche Diagnostics, Mannheim, Germany). The lysates were centrifuged at 14,000xg for 20 min at 4°C. The protein content of the supernatant was determined with the Bradford protein assay kit (Sigma Aldrich, U.S.A.) as recommended by the manufacturer.

### 2.11. Western Blotting

For Western blotting, proteins obtained were subjected to SDS-PAGE on 12.5% gel. The proteins were then transferred to polyvinylidene fluoride membranes (PVDF) [Thermo Fisher Scientific, U.S.A]. Subsequently, the membrane was blotted with different antibodies like mouse monoclonal cytochrome c (1 : 300; Santa Cruz Biotechnology) and mouse monoclonal beta actin (1 : 1000; Santa Cruz Biotechnology). The immune complexes were visualized using enhanced chemiluminescence (ECL) method.

### 2.12. Quantitative Real-Time PCR

Messenger RNA (mRNA) expressions of antiapoptotic protein of the B cell lymphoma 2 (BCL-2) and proapoptotic protein BCL-2-associated X protein (BAX), C/EBP-homologous protein (CHOP), and glucose-regulated protein 78 (GRP78) in isolated hepatocytes were measured by quantitative real-time PCR (qRt-PCR) using specific primers in thermal cycler system (applied biosystem 7300, U.S.A). Messenger RNA expression was normalized using housekeeping gene *β* actin. The primers used were as follows:

- BCL-2 (forward): 5′-GACAGAAGATCATGCCGTCC-3′

    (reverse): 5′-GGTACCAATGGCACTTCAAG-3′

(b) BAX (forward): 5′-GCGTCCACCAAGAAGCTGAG-3′

    (reverse): 5′-CCCCAGTTGAAGTTGCCATCA-3′

(c) CHOP (forward): 5′-GCGACAGAGCCAGAATAACA-3′

     (reverse): 5′-GATGCACTTCCTTCTGGAACA-3′

(d) GRP78 (forward): 5′-CTGAGGCGTATTTGGGAAAG-3′

     (reverse): 5′-TCATGACATTCAGTCCAGCAA-3′

(e) *β*-Actin (forward): 5′TGGAATCCTGTGGCATCCATGAAAC-3′

     (reverse): 5′AAAACGCAGCTCAGTAACAGTCCG-3′

### 2.13. Statistical Analysis

Data are expressed as mean ± SD. Statistical analysis was performed using SPSS software (SPSS 7.5 for windows; SPSS Inc., Chicago, IL). Intergroup differences were analyzed using one-way ANOVA. A *p* value of <0.05 was considered as statistically significant.

## 3. Results

### 3.1. Arsenic Induces ROS Formation at an Early Hour

**Figure 1:**
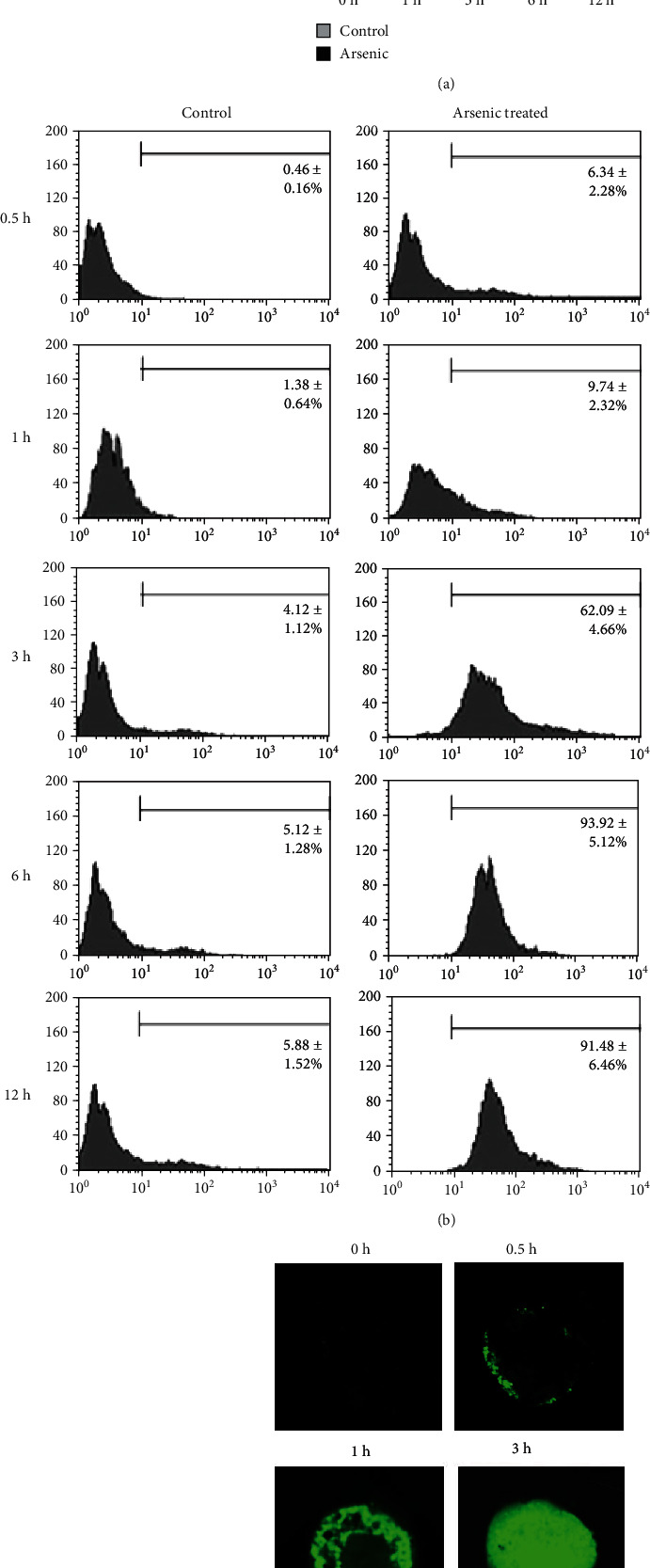
Arsenic-induced ROS formation in primary cultured mouse hepatocytes using DCF-DA. The results are expressed as mean ± SD independent experiments of 6 independent experiments. (a) Time-dependent arsenic (10 *μ*M)-induced ROS in the hepatocytes was quantified as relative fluorescence intensity using multiplate reader. ^∗^*p* < 0.01 vs. control and ^∗∗^*p* < 0.001 vs. control. (b) The histogram represented ROS level produced by mouse hepatocytes in flow cytometry analysis with or without arsenic (10 *μ*M) treatment at different time points. (c) Microscopically, intracellular ROS was evaluated at different time periods after staining with DCF-DA using confocal microscope. The representative figure showed that at 30 min of arsenic treatment, ROS generation was started from the peripheral region of a mouse hepatocyte and gradually progressed to the center of the cell with progression of time (40x magnifications). The figure is representative photomicrograph of 3 independent experiments.

Primary cultured mouse hepatocytes were labeled with DCF-DA and were used to evaluate the extent of intracellular ROS generation at different time intervals in presence or absence of arsenic. Hepatocytes in presence of arsenic expressed significant increase in ROS production from 1 h (2.5 ± 0.43 × 10^3^ RFU; *p* < 0.01) compared to control (hepatocytes without arsenic treatment) which maintained relatively stable and low ROS production of (1.2 ± 0.25) to (1.4 ± 0.25) × 10^3^ RFU during the experimental period (Figure 1(a)). The intracellular ROS during arsenic treatment within the hepatocytes increased 14-fold compared to control at 3 h and gradually decreased to 9.5-fold after 12 h of arsenic exposure (Figure 1(a)).

Time-dependent intracellular ROS formation within hepatocytes during arsenic exposure was confirmed by flow cytometry data using BD FACS Calibur (Figure 1(b)). Next, we evaluated arsenic-induced intracellular ROS within hepatocytes using confocal microscope after staining with DCF-DA. No specific green fluorescence pattern was noted in control cells. The appearance of green DCF fluorescence at the periphery of a cell (cell membrane) was first noticed at 30 min of arsenic exposure that subsequently progressed towards the central part of the cells with time (Figure 1(c)).

### 3.2. Early Phase of ROS Generation Is NADPH Oxidase Dependent

To identify the primary source of ROS within the isolated mouse hepatocytes, we exposed primary cultured mouse hepatocytes to 10 *μ*M arsenic following pretreatment with or without 10 *μ*M DPI or 10 *μ*M CsA 30 min before arsenic exposure. Arsenic-induced ROS formation was measured at 1 and 3 h using a multimode plate reader.

**Figure 2:**
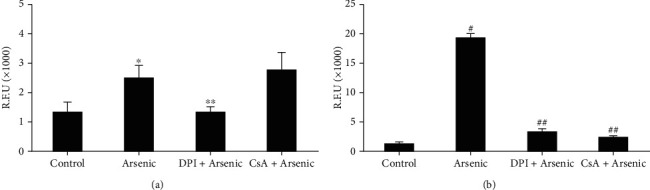
Effect of DPI, a NOX inhibitor, and cyclosporin, an inhibitor of mitochondrial permeability transition (MPT), on arsenic-induced ROS generation in primary cultured mouse hepatocytes. Cells were pretreated with either DPI or cyclosporin (10 *μ*M) at least 30 min before the treatment of 10 *μ*M arsenic. (a) After 1 h of arsenic treatment, quantitative assessment of ROS production was measured by multimode plate reader. ^∗^*p* < 0.01 vs. control; ^∗∗^*p* < 0.004 vs. arsenic; (b) after 3 h of arsenic treatment, quantitative assessment of ROS production was measured by multimode plate reader. ^#^*p* < 0.001 vs. control; ^##^*p* < 0.001 vs. arsenic.

Addition of DPI markedly reduced arsenic-induced intracellular ROS within the hepatocytes at 1 h that was comparable to that of control (Figure 2(a)). Treatment of CsA prior to arsenic exposure did not show any inhibition of ROS at 1 h (Figure 2(a)) suggesting the fact that ROS generated due to arsenic exposure may be associated with NADPH oxidase but not from the mitochondrial sources at an early hour of arsenic exposure. Cyclosporin A was effective in preventing arsenic-induced ROS formation at 3 h (Figure 2(b)).

### 3.3. Arsenic-Induced ROS Generation and Lysosomal Membrane Permeabilization

**Figure 3:**
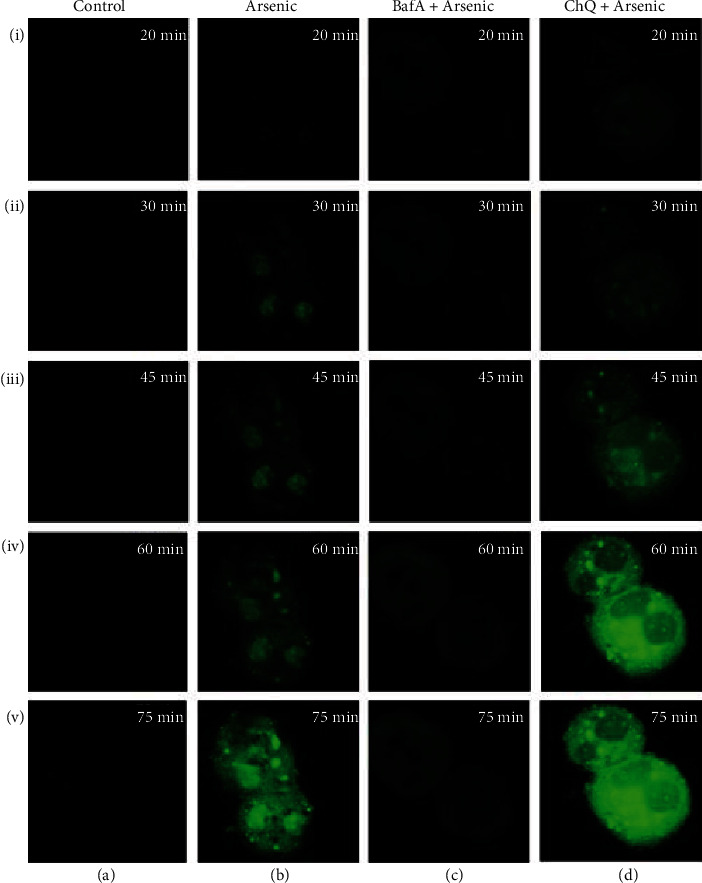
Alteration of arsenic-induced LMP evaluated by increase in green fluorescence as observed by confocal microscope using AO relocation technique in presence or absence of LMP inducer (BafA) and inhibitor (ChQ) at different time intervals. Control (a) and arsenic (b) exposed primary cultured mouse hepatocytes were preincubated with or without BafA (c) and ChQ (d) (magnification of all the figures are 40x). The figure is representative photomicrographs of 3 independent experiments. LMP was not observed in control hepatocytes since no significant green fluorescence was noted from 20 min to 75 min (ia-va). Arsenic exposure of mouse hepatocytes for 30 min only exhibited the appearance of green fluorescence which eventually increased with time (ib-vb). Pretreatment with BafA prior to arsenic treatment significantly prevented LMP as evidenced by negligible green fluorescence (ic-vc). LMP was further exacerbated following pretreatment with ChQ before arsenic treatment as depicted from 30 min of arsenic exposure in a time-dependent manner (id-vd).

Since arsenic have been reported to accumulate in lysosomes [21, 22], we hypothesized that arsenic-induced lysosomal rupture may be an early event in liver cell injury. Arsenic-induced lysosomal membrane leakage leading to LMP was assessed using AO relocation method. Acridine orange is a lysosomotropic lipophilic fluorochrome which preferentially distributes within lysosome. During lysosomal membrane leakage, AO accumulates in the cytoplasm and displays green fluorescence when excited by blue light. Next, we pretreated hepatocytes either with BafA or with ChQ 30 min prior to arsenic exposure, to inhibit and induce LMP, respectively. Untreated control cells hardly showed any green fluorescence (Figure 3 va), while at 30 min, only green fluorescence in the cytosol appeared in arsenic-exposed hepatocytes (Figure 3 iib), and the increase in green fluorescence gradually with time due to the release of AO from leakage of lysosomes was observed (Figure 3 vb).

Pretreatment with 10 *μ*M of BafA 30 min prior to arsenic exposure prevented LMP even at 1 h (Figure 3 ivc) while 20 *μ*M ChQ pretreatment induced LMP at 30 min only as observed from the green fluorescence of AO (Figure 3 id) which gradually increased with time in a significant manner as compared to arsenic-treated group (Figure 3 vd).

To confirm our hypothesis that LMP preceded arsenic-induced ROS generation, hepatocytes were pretreated either with BafA or with ChQ 30 min prior to arsenic exposure.

**Figure 4:**
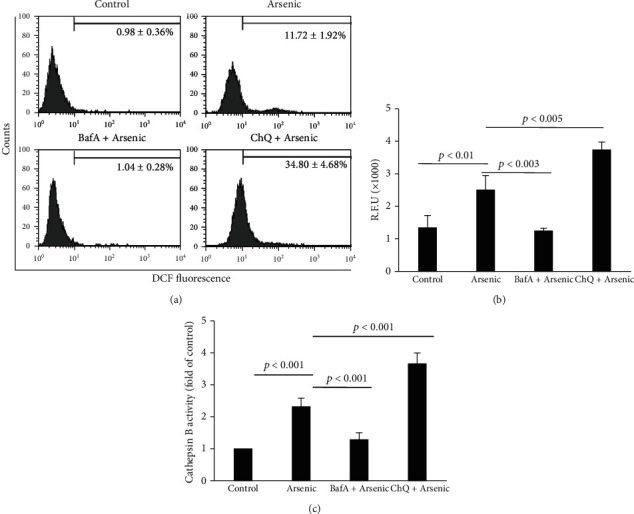
Effect of BafA and ChQ on arsenic-induced ROS formation and cathepsin B activity. (a) Representative flow cytometric analysis of arsenic induced ROS at 1 h using DCF-DA. (b) Quantification of ROS using multiplate reader at 1 h. The results indicate the mean ± SD of 6 independent experiments. (c) Cathepsin B activity from the cytosol of control and arsenic-exposed hepatocytes pretreated with or without BafA and ChQ. The results are representative of 6 identical experiments.

ChQ treatment accelerated ROS production as revealed by flow cytometry (Figure 4(a)). ChQ pretreatment prior to arsenic exposure increased the number of ROS producing cells by approximately 35% compared to only 12% arsenic-treated cells at 1 h (Figure 4(a)). There was a significant increase in the intensity of DCF fluorescence as well in ChQ pretreated group suggesting that LMP aggravated the ROS production at 1 h within the hepatocytes due to arsenic exposure (Figure 4(b)). Pretreatment with BafA prior to arsenic exposure for 1 h not only prevented LMP as observed from the AO staining of the primary cultured hepatocytes (Figure 3 ivc) but also cytosolic ROS formation as evidenced by flow cytometry (Figure 4(a)) as well as fluorescence spectrophotometer (Figure 4(b)).

Since LMP is associated with cytosolic translocation of cathepsins and to validate the findings of LMP, we estimated cathepsin B activity at 1 h in cytosolic fraction from the arsenic-treated hepatocytes in the absence or presence of BafA or ChQ. Cathepsin B activity measured from arsenic-treated hepatocytes indicated a 2.3-fold increase (Figure 4(c)). BafA-pretreated arsenic-exposed hepatocytes exhibited insignificant cytosolic cathepsin B activity that was comparable to that of the control. However, treatment of 20 *μ*M ChQ along with arsenic aggravated the cytosolic accumulation of cathepsin B that was found to be 1.6-fold higher as compared to arsenic treatment alone.

### 3.4. Onset of MPT and Mitochondrial Depolarization after Arsenic-Induced ROS Formation

**Figure 5:**
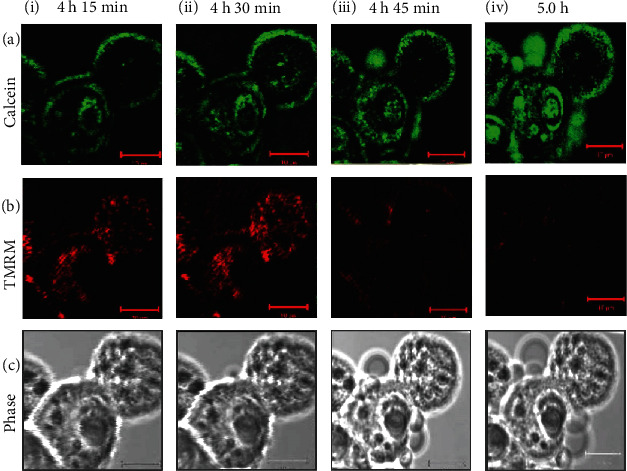
Onset of mitochondrial permeability transition (MPT) in isolated mouse hepatocytes due to arsenic exposure. Green fluorescence of calcein (a), red fluorescence of TMRM (b), and phase images (c) of the unfixed hepatocytes were imaged simultaneously by confocal microscopy, and images were represented after 4 h 15 min (i); 4 h 30 min (ii); 4 h 45 min (iii), and 5 h (iv) of arsenic exposure. In the baseline images, green fluorescence of calcein was limited to the cytosol and nucleus, whereas red fluorescence of TMRM was observed in the mitochondria. Till 4 h 15 min of arsenic exposure, the baseline images remained unchanged (i). After 4 h and 30 min of arsenic exposure, TMRM fluorescence was increased (iib) indicating hyperpolarization of the mitochondria, whereas there was no significant change of green fluorescence of calcein as shown in the image (iia). After 4 h and 45 min, calcein began to redistribute from the cytosol into the mitochondria as evident by increase distribution of green fluorescence, indicating onset of MPT (iiia) with simultaneous membrane blebbing as shown by the phase image (iiic), and mitochondria lost most of the TMRM red fluorescence (iiib). After 5 h, TMRM red fluorescence almost disappeared rather weak, and one cell lost TMRM fluorescence completely (ivb). Increase in cell membrane blebs was seen in the phase image (ivc) as well as calcein fluorescence was almost uniformly distributed (iva) indicating the completion of MPT. Scale bar (−−) is 10 *μ*m.

To observe the changes of MPT caused by arsenic treatment, hepatocytes were pretreated with TMRM and calcein before arsenic exposure. We did not notice spontaneous MPT within 8 h of observation in control hepatocytes. Initially, the hepatocytes showed green calcein fluorescence that was confined to the cytosol and nuclei (Figure 5 ia), leaving the dark voids corresponding to mitochondria. The mitochondria were stained with bright red-fluorescing TMRM, which are typical of normally polarized mitochondria in hepatocytes (Figure 5 ib). No alterations in the TMRM and calcein fluorescence patterns were observed till 4 h of arsenic treatment. The red fluorescence of TMRM was found to be intensified (Figure 5 iib) without any significant change in calcein fluorescence at 4 h 30 min (Figure 5 iia) indicating hyperpolarization of the mitochondrial membrane. A small cellular bleb appeared at that point of time. Confocal microscopy exhibited the onset of MPT at 4 h and 45 min of arsenic treatment, as indicated by the redistribution of calcein fluorescence into the mitochondrial voids (Figure 5 iiia) with decrease in TMRM fluorescence (Figure 5 iiib) followed by increase in blebbing of the cell membrane (Figure 5 iiic).

At the 5^th^ h, complete mitochondrial depolarization was observed as indicated by the further redistribution of calcein into the mitochondria of the 2 cells observed, filling the dark voids as well as absolute loss of TMRM fluorescence (Figure 5 iv).

### 3.5. ROS-Induced Cell Death in Arsenic-Treated Cells

**Figure 6:**
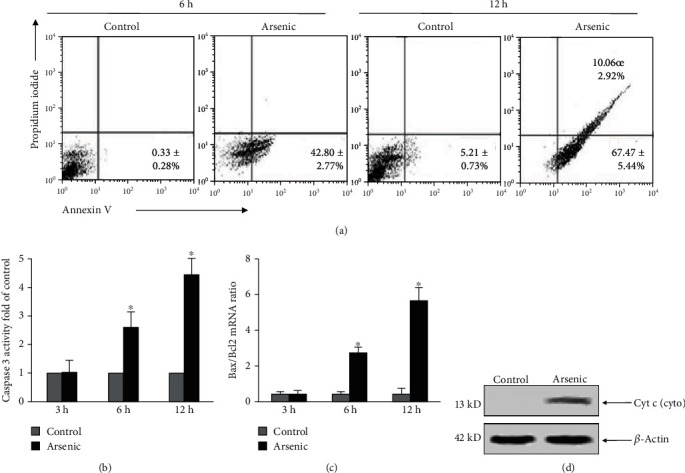
Mode of cell death due to arsenic exposure at a dose of 10 *μ*M was assessed by Annexin V and propidium iodide staining. (a) Flow cytometric quantification of the percentage of dead cells at 6 h and 12 h of arsenic exposure using Annexin V and PI staining. The data are representative photomicrographs of 6 independent experiments. (b) Caspase 3 activity was quantified by ELISA at different hours of arsenic exposure. The results are representative of 6 independent experiments. ^∗^*p* < 0.001 vs. control. (c) mRNA expression of the proapoptotic protein Bax and antiapoptotic protein Bcl2 was quantified using real-time PCR and plotted graphically. ^∗^*p* < 0.001 vs. control. (d) Representative Western blot of cytochrome c from the cytosol of control and arsenic-treated hepatocytes.

Arsenic-induced hepatocyte death as assessed by PI and Annexin-V staining was not observed till 6 h. After 6 h of incubation at a dose of 10 *μ*M, arsenic caused apoptosis of 43% of hepatocytes compared to less than 1% in control group as observed by flow cytometry (Figure 6(a)).

However, prolonged exposure to arsenic for 12 h significantly increased the percentage of apoptotic cells where a dual positivity of the cells was observed when stained with PI and Annexin V indicating early apoptosis as well as late apoptosis (Figure 6(a)). After treatment of hepatocytes with arsenic alone at different time points, caspase 3 activity was determined to further assess the role of caspase 3 activation in arsenic treated cells. Arsenic treatment showed an increase of 2.5 ± 0.58 and 4.46 ± 0.55 fold in caspase 3 activity at 6 and 12 h, respectively (Figure 6(b)).

To further investigate the typical features of apoptosis, the mRNA levels of BAX and BCL-2 were measured in hepatocytes treated with arsenic for 3, 6, and 12 h.  Figure 6(c) demonstrated a significantly elevated ratio of BAX/BCL-2 in hepatocytes with increase in the duration of arsenic exposure. No remarkable difference in the ratio of BAX/BCL-2 was evident at 3 h of arsenic treatment compared to control group (Figure 6(c)) suggesting the fact that induction of apoptosis mediated by arsenic occurred through increased expression of proapoptotic protein BAX and alternation of the BAX/BCL-2 ratio.

Further to understand, whether ER stress during arsenic exposure have any role in cellular apoptosis, we focused on ER stress. Of many proteins of interest, the chaperone protein, GRP78, is considered as a common marker of ER stress. We carried out mRNA expression of GRP78 by real-time PCR. We observed that mRNA expression of GRP78 was 1.27 ± 0.46 fold and 3.73 ± 0.93 higher than that of control groups in 3 and 6 h, respectively. However, at 12 h there was a decrease in mRNA expression of GRP78 (0.67 ± 0.15). Subsequently, we also studied the mRNA expression pattern of CHOP, a transcriptional factor, whose expression was also found to be high by ER stress. Expression of CHOP mRNA also followed a similar pattern (1.18 ± 0.08 at 3 h and 2.94 ± 0.64 at 6 h of arsenic exposure) like that of GRP78. At 12 h of arsenic exposure, CHOP expression was also decreased to 0.75 ± 0.3.

As a result of MPT, cytochrome c was found to be released from mitochondria to the cytosol as assessed by Western blotting. Cytochrome c was readily detectable in the cytosol of arsenic-treated cells after 6 h of arsenic exposure (Figure 6(d)), whereas no sign of cytochrome c translocation was noted in the control hepatocytes (Figure 6(d)).

## 4. Discussion

In the present study, increased ROS formation, as observed in response to arsenic exposure in isolated mouse hepatocytes, originates as a consequence of activation of NOX, LMP, and translocation of cathepsin B to the cytosol. All these cellular events subsequently stimulate the mitochondria to further amplify ROS production altering the permeability transition leading to hepatocytes death. Apoptosis has been identified as the major mechanism of cell death following arsenic exposure. In this present study, we demonstrate leakage of lysosomal membrane to be an early event triggering mitochondrial dysfunction followed by caspase activation which in turn induces apoptosis of hepatocytes in response to arsenic suggesting a functional link between lysosomes and mitochondria.

Accumulating evidences suggest that lysosome-mediated apoptosis plays a crucial role in cell death process in response to numerous stimuli [19, 23, 24]. Although several agents have been demonstrated to be involved in the induction of LMP and apoptosis [25], we report in this paper LMP as the initiator of ROS generation after arsenic exposure since LMP is detectable in isolated mouse hepatocytes within 30 min after arsenic treatment resulting in early release of cathepsins to the cytosol.

Although our data show only 2.3-fold increase in the cathepsin B activity at 1 h of arsenic exposure, yet we are unaware of the threshold of cathepsin B required for executing the apoptotic pathway. LMP inhibitor BafA delays arsenic-induced LMP and decreases cathepsin B activity. On the other hand, pretreatment with lysosomotropic agent ChQ enhances the cytotoxicity of arsenic resulting in significant LMP as well as elevated cathepsin B activity. BafA is also shown to protect cells from arsenic induced oxidative toxicity by inhibiting intracellular ROS generation suggesting that LMP precedes enhanced ROS generation in primary mouse hepatocytes.

Mitochondria serve as the most essential organelle in the process of apoptosis [26, 27]. In our present study, we have observed that the mitochondria maintain its normal integrity till 4 hours after which it was initially hyperpolarized before the onset of MPT. One possible explanation for this may be that the ROS generated during the course of metabolism of arsenic reaches its peak when the antioxidant defence system fails to counteract the increased ROS threat. This increased ROS may alter fluidity of the inner membrane favoring pore formation. This finding is highly in accordance with the notion that LMP-induced ROS can trigger a transient increase in mitochondrial ROS production via ROS activation of the MPT pore, a phenomenon termed as ROS-induced ROS release (RIRR) [28]. Primary mouse hepatocytes undergo apoptosis after exposure to arsenic for 6 and 12 h in a time-dependent manner as evidenced by detection through fluorescein Annexin V-FITC/PI double labeling. Apoptosis of hepatocytes is associated with caspase 3 activation, downregulation of BCL-2, and increase in BAX. Antiapoptotic protein, BCL-2, plays an important role in the regulation of MPT and inhibiting apoptotic cell death [29]. The proapoptotic protein BAX activates MPT through interaction with the voltage-dependent anion channel [30] and/or the adenine nucleotide translocator [31] in cells and isolated mitochondria. In our study, the decrease in BCL-2 expression with subsequent increase in BAX expression may also contribute to the MPT in addition to ROS generation. Furthermore, overexpression of BAX also enhances the production of ROS within the cell [32]. Thus, we can presume that in our study, decrease expression of Bcl-2 facilitates Bax translocation to the mitochondria, thereby causing MPT. It is also documented that LMP can also directly cause alteration of MPT [23].

**Figure 7:**
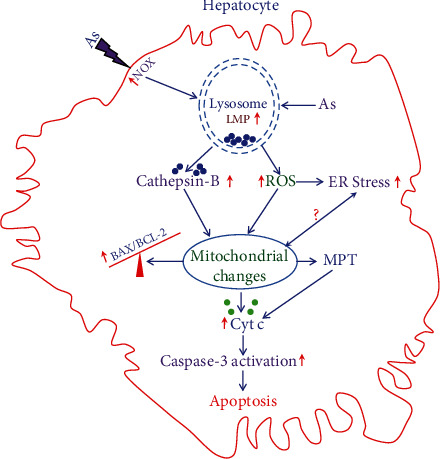
Schematic diagram of the proposed cellular events during arsenic exposure to hepatocytes. Arsenic exposure to hepatocyte causes activation of plasma membrane-bound NOX and generation of ROS in the cell. Both ROS and arsenic cause leakage of lysosomal membrane leading to LMP resulting in release of cathepsin B and increased ROS generation. Both cathepsin B and increased ROS in turn cause mitochondrial changes leading to rapid onset of MPT that is upstream of cytochrome c release as well as increase in BAX and BCL-2 ratio. Cytochrome c further promotes the effector caspase activation leading to apoptotic death of hepatocytes. Increased ROS causes induction of ER stress at a later time period, and the role of ER stress in mitochondrial functional changes in our study is debatable.

The possible mechanisms related to hepatocytes death during arsenic exposure are shown in Figure 7. The initial events arise from membrane bound NOX and intracellular ROS that cause disturbance of homeostasis of lysosomes and induction of LMP. Leakage of membrane of lysosomes facilitates release of cathepsin B as well as increases cytosolic ROS formation within hepatocytes. Both cathepsin B and ROS initiate rapid onset of MPT and release of proapoptotic factors from mitochondria resulting activation of caspase 3 and cell death. Increase intracellular ROS also activates ER stress at a later time point possibly after onset of MPT.

In summary, our results demonstrate that ROS generation due to LMP is a key factor in arsenic-induced apoptosis. The lysosomal ROS stimulates mitochondrial production of ROS, where it is amplified thereby causing mitochondrial injury. The abrogation of ROS generation not only prevents the alteration of MPT but also apoptosis as well. These findings now provide a better elucidation of the mechanisms involved in arsenic-induced apoptosis and should lead to better understanding of the possible way of preventing arsenic-induced liver cell injury.

## Data Availability

The data used to support the findings of this study are included within the article.

## Abbreviations

LMP: Lysosomal membrane permeabilization
MPT: Mitochondrial permeability transition
ROS: Reactive oxygen species
DCFH-DA-2,7: Dichlorofluorescein diacetate
TMRM: Tetramethylrhodamine methyl ester
NOX: NADPH oxidase
ChQ: Chloroquine
BafA: Bafilomycin A
DPI: Diphenyliodonium
CsA: Cyclosporin A
PI: Propidium iodide
BCL-2: B cell lymphoma 2
BAX: BCL-2 associated X protein
CHOP: C/EBP-homologous protein
GRP78: Glucose-regulated protein 78
ER: Endoplasmic reticulum
qRT-PCR: Quantitative real-time polymerase chain reaction
SD: Standard deviation.
